# Ultraconserved element uc.333 increases insulin sensitivity by binding to miR-223

**DOI:** 10.18632/aging.103020

**Published:** 2020-04-17

**Authors:** Yang Zhang, Jingyu Sun, He Yao, Yajun Lin, Jie Wei, Gang Hu, Jun Guo, Jian Li

**Affiliations:** 1 Peking University Fifth School of Clinical Medicine, Beijing, China; 2 The Key Laboratory of Geriatrics, Beijing Institute of Geriatrics, Beijing Hospital, National Center of Gerontology, National Health Commission, Institute of Geriatric Medicine, Chinese Academy of Medical Sciences, Beijing, China

**Keywords:** insulin resistance, uc.333, NAFLD, miR-223, FOXO1

## Abstract

Insulin resistance (IR) contributes to diabetes and aging. Ultraconserved elements (UCEs) are a class of long noncoding RNAs (lncRNAs) that are 100% conserved in humans, mice, and rats. We identified the lncRNA uc.333 using an lncRNA microarray and then used quantitative real-time polymerase chain reaction to analyze its expression in the livers of nonalcoholic fatty liver disease (NAFLD) patients, db/db mice, high-fat diet–fed mice, IL-6-treated mice, and TNF-α-treated mice. The underlying mechanisms of uc.333 in IR were investigated using fluorescence in situ hybridization, Western blot, and miRNA microarray analyses. The results revealed that uc.333 expression was decreased in liver tissues from NAFLD patients and treated mice. Furthermore, overexpression of uc.333 decreased IR, whereas knocking down uc.333 increased IR. We also confirmed that uc.333 binds to miR-223 and that the levels of miR-223 were increased in the livers of patients and treated mice. These findings showed that uc.333 improves IR by binding to miR-223; thus, uc.333 may be a useful target for the treatment and prevention of IR.

## INTRODUCTION

Insulin resistance (IR) is a leading cause of more than 90% all diagnosed cases of diabetes and metabolic syndromes [1, 2]. It is a major contributor to a series of pathogenesis reactions, including atherosclerosis, hypertension, alcoholic fatty liver disease (AFLD), and nonalcoholic fatty liver disease (NAFLD) [1, 3]. IR is an epidemic condition prevalent worldwide and has been shown to strongly correlate with the inflammatory factors that cause abnormal glucose metabolism in hepatocytes and vessel walls, [4] thereby leading to liver injury and atherosclerosis [5, 6].

Emerging evidence shows that long noncoding RNAs (LncRNAs) regulate IR [7]. Among LncRNAs, the ultraconserved noncoding RNAs (ucRNAs), termed ultraconserved elements (UCEs), include 481 members and transcribe across the human, mouse, and rat genomes with 100% conservation. They are frequently located in gene fragile sites and cancer-associated genomic regions [8, 9]. Abnormal expression of ucRNAs is widely identified in many types of tumors [10, 11]. For example, uc.416 promotes the progression of gastric cancer [12]. Additionally, our previous study demonstrated that aberrant expression of UCR372 (uc.372) in the livers of NAFLD patients regulated hepatic steatosis through the inhibition of miR-195/miR-4668 maturation. However, the function of ucRNAs in IR is still unclear [13].

In this study, we demonstrate how uc.333 works and regulates IR in the livers of NAFLD patients and experimental animals. This study is the first to determine the expression and functional role of uc.333 in IR and describes the role ucRNAs play in IR progression.

## RESULTS

### The level of uc.333 was reduced in the livers of the high-fat diet (HFD)-fed mice and db/db mice

To assess transcribed ultraconserved regions (T-UCRs), we profiled lncRNA expression using a microarray with liver tissue from NAFLD patients and controls. The findings from lncRNA-wide expression profiling identified 8 ucRNAs, which represented 1.6% of all ucRNAs analyzed, whose expression was aberrantly decreased by ~4.9- to ~45.4-fold in the livers of NAFLD patients compared with those of controls.

**Figure 1:**
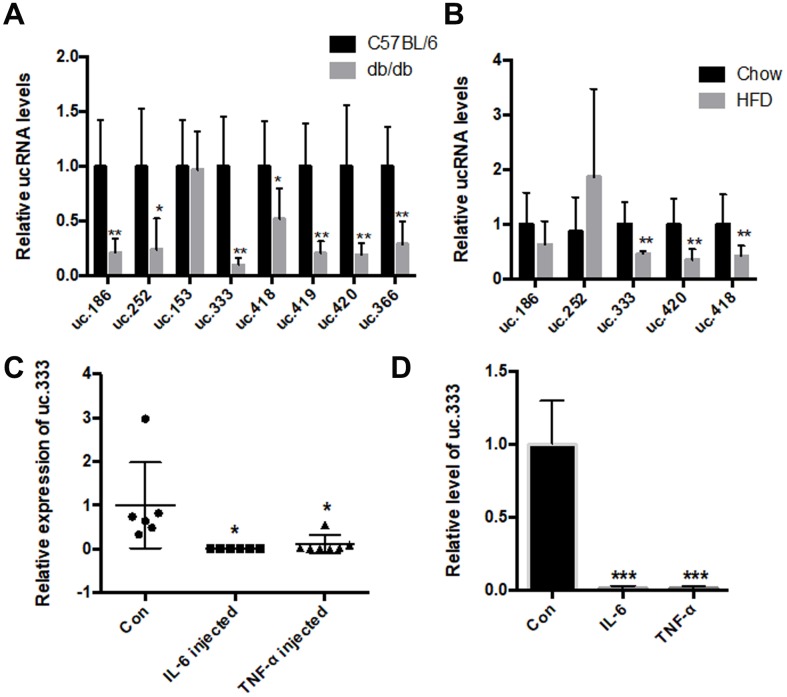
**The level of uc.333 was reduced in the livers of the HFD-fed mice and db/db mice.** (**A**) Expression of ucRNAs in the liver of db/db mice analyzed using real-time PCR (n = 6). (**B**) Expression of ucRNAs in the liver of HFD-fed mice analyzed using real-time PCR (n = 6). (**C**) Expression of uc.333 in liver of 10-week-old mice injected with IL-6 16 μg /mL and TNF-α 16 μg /mL for 7 days. (**D**) Expression of uc.333 in HepG2 cells induced with 20 nM TNF-α and 20 nM IL-6. Data are mean ± SEM; *P < 0.05; **P < 0.01; ***P < 0.001 vs. control group (Student’s t test).

We then verified the expression of the 8 T-UCRs using real-time polymerase chain reaction (PCR) in the liver tissues of db/db mice and controls, and uc.186, uc.252, uc.333, uc.418, uc.419, uc.366, and uc.420 were confirmed to be significantly downregulated (Figure 1A). To determine whether these T-UCRs were specifically altered metabolically or affected only because of the diabetic condition, we tested T-UCR expression in the livers of HFD-fed mice and found that the levels of uc.333, uc.420, and uc.418 were decreased in the livers of the HFD-fed mice compared with chow-diet mice and, specifically, uc.333 was decreased the most in both the db/db and HFD mice (Figure 1B). Hence, we focused on uc.333 for further study, noting that it is a part-exonic UCR located on chromosome 11 with 273 nt in the human genome.

We tested the level of uc.333 in the mice that had been injected with interleukin-6 (IL-6) and tumor necrosis factor-α (TNF-α) using a subcutaneous implantation pump. Our data showed that the level of uc.333 was significantly decreased in the livers of both the IL-6-treated and TNF-α-treated mice (Figure 1C). To determine whether uc.333 was relevant to the metabolic milieu in vitro, we assessed the expression of uc.333 in human HepG2 hepatocytes stimulated by 10 nmol/L TNF-α and 10 nmol/L IL-6 for 24 h. As shown in Figure 1D, IL-6 and TNF-α effectively downregulated uc.333 expression in the HepG2 cells.

### Distribution of uc.333 in various tissues and hepatocytes

**Figure 2:**
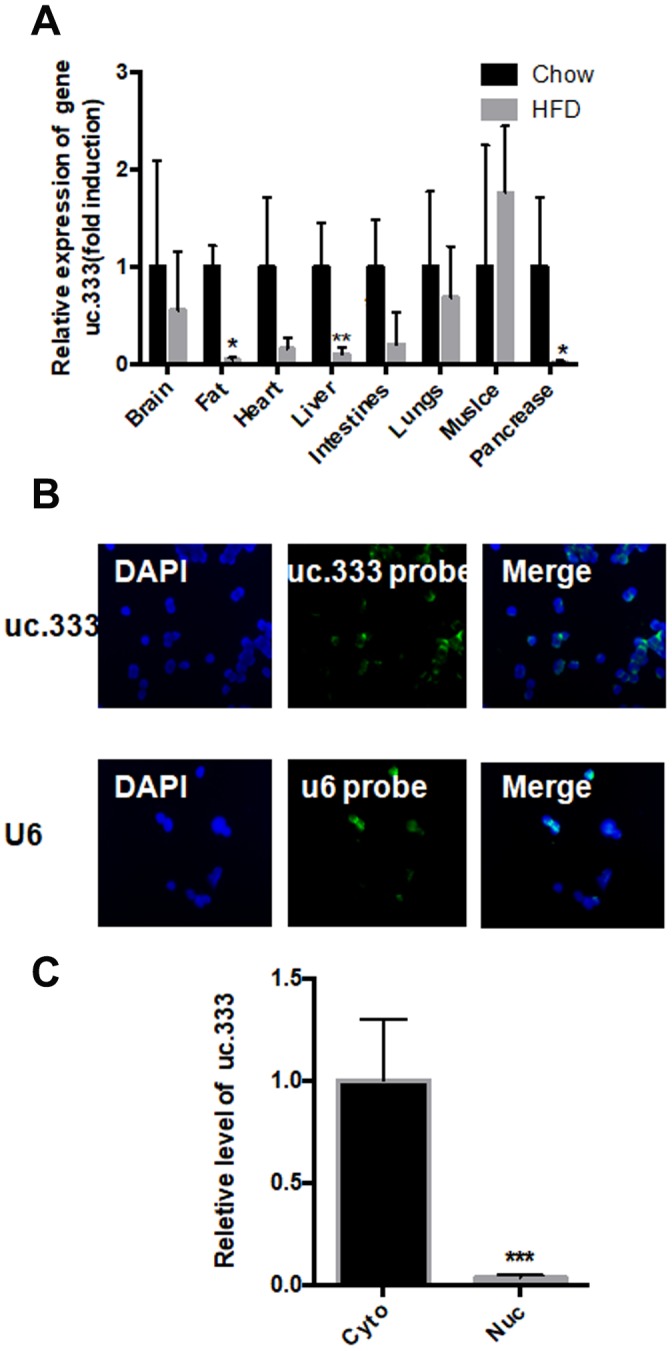
**Distribution of uc.333 in various tissues and in hepatocytes.** (**A**) Distribution of uc.333 in various tissues of HFD-fed mice (n = 5). (**B**) Representative images of FISH detecting endogenous lncRNA uc.333 molecules (green) in HepG2 cells. Nucleus (blue) was stained with DAPI. Scale bar, 25 μm. (**C**) Cellular fractionation assay in HepG2 was performed using quantitative real-time PCR using a specific cytosol control (tubulin) and a specific nuclear control (gene histone). Data are mean ± SEM; *P < 0.05; **P < 0.01; ***P < 0.001 vs. control group (Student’s t test).

Next, we determined the distribution of uc.333 in various tissues. We found that uc.333 was widely expressed in mouse organs (Figure 2A). The level of uc.333 expression was significantly decreased in metabolic-related tissues, including fat and liver, implicating uc.333 as a potential metabolic regulator in vivo. These findings prompted us to determine the underlying mechanism by which uc.333 regulated the progression of IR. The results from RNA fluorescence in situ hybridization (RNA-FISH) demonstrated that uc.333 was predominantly located in the cytoplasm (Figure 2B). High uc.333 probe expression was also detected in the cytoplasm (Figure 2C). We also assessed the relative level of uc.333 in the cytoplasm and nucleus of HepG2 cells through RNA fractionation. We observed that uc.333 was predominantly located in the cytoplasm, suggesting that uc.333 might exert its biological function in the cytoplasm.

### Overexpression of uc.333 decreased IR

**Figure 3:**
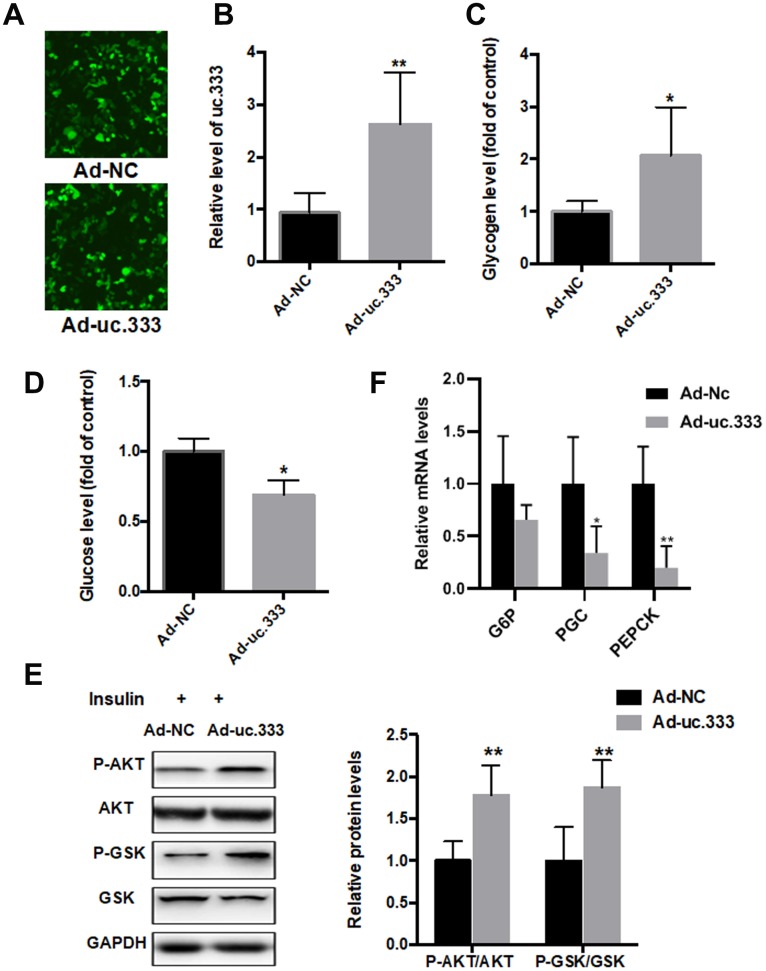
**Overexpression of uc.333 ameliorated IR.** (**A**) Immunofluorescence assay showed that Ad-NC or Ad-uc.333 transfected into HepG2 cells with equal transfection efficiency.(**B**) Level of uc.333 in the HepG2 cells transfected with Ad-uc.333 or Ad-NC for 48 h. (**C**) Glycogen content in HepG2 cells after transfection with Ad-uc.333 or Ad-NC for 48 h. (**D**) Expression of glucose in HepG2 cells transfected with Ad-uc.333m or Ad-NC for 48 h. (**E**) Protein levels of P-AKT, AKT, P-GSK, GSK, and GAPDH in HepG2 cells transfected with Ad- uc.3333 or Ad-NC for 48 h, as analyzed using Western blot. (**F**) The mRNA levels of G6Pase, PGC-1α and PEPKC were also decreased after overexpression of uc.333 compared with that of Ad-NC Data are mean ± SEM; *P < 0.05; **P < 0.01; ***P < 0.001 vs. control group (Student’s t test).

To determine whether uc.333 increases insulin sensitivity and thus decreases IR, we overexpressed uc.333 in HepG2 cells. Immunofluorescence assay showed that Ad-NC or Ad-uc.333 transfected into HepG2 cells with equal transfection efficiency (Figure 3A). The results from the reverse transcriptase PCR (RT-PCR) analysis demonstrated that uc.333 expression was substantially upregulated in the HepG2 cells transfected with Ad-uc.333 (Figure 3B). As shown in Figure 3C, overexpression of uc.333 in the HepG2 cells increased glycogen contents transfected with Ad-uc.333 (Figure 3C). In addition, intracellular glucose content was decreased in the HepG2 cells overexpressing uc.333 (Figure 3D). We also examined the effect of uc.333 on PI3K/AKT/GSK signaling, which is a key regulator of IR. In HepG2 cells, overexpression of uc.333 significantly increased the phosphorylation levels of PI3K/AKT/GSK (Figure 3E). The mRNA levels of G6Pase, PGC-1α and PEPKC were also decreased after overexpression of uc.333 compared with that of Ad-NC (Figure 3F).

### Inhibition of uc.333 promoted IR

**Figure 4:**
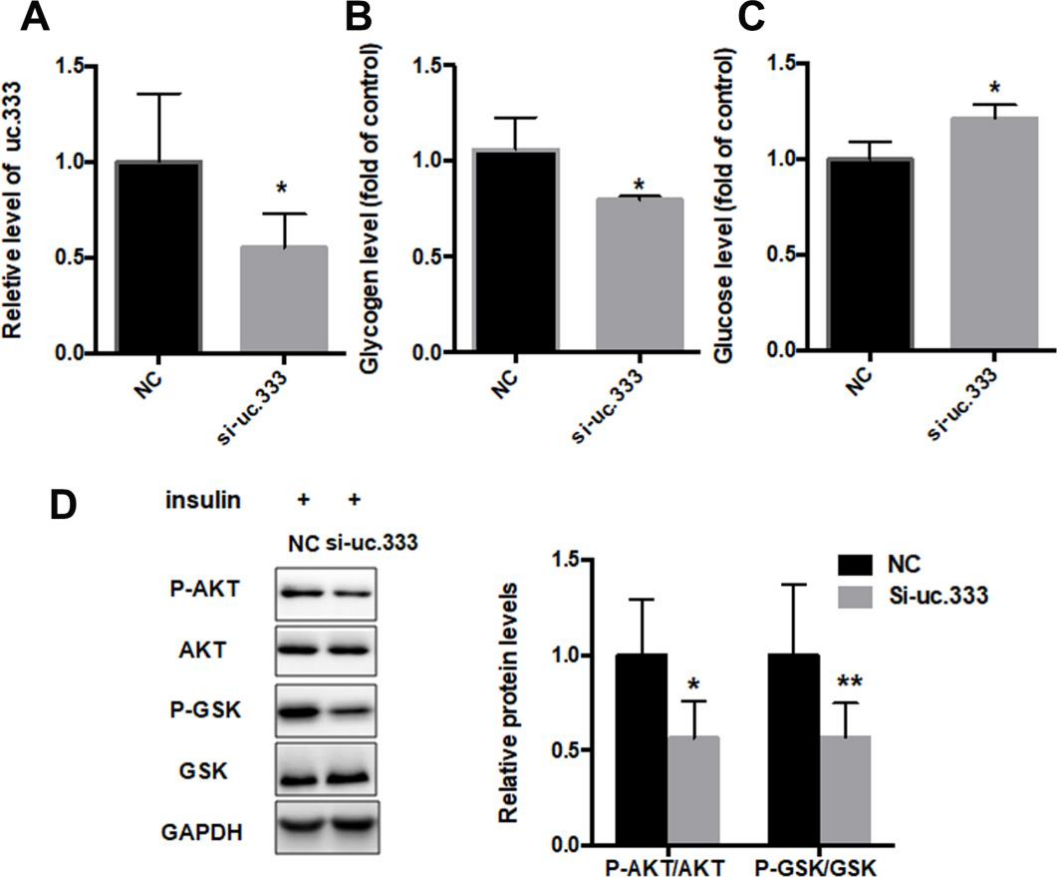
**Inhibition of uc.333-promoted IR.** (**A**) The results of quantitative RT-PCR analysis for the expression of uc.333 in HepG2 cells transfected with negative control (NC) or siRN. (**B**) Determination of glycogen content in HepG2 cells after transfection with si-uc.333 or NC for 48 h. (**C**) Expression of glucose in HepG2 cells transfected with si-uc.333m or NC for 48 h. (**D**) Expression of P-AKT, AKT, P-GSK, GSK, and GAPDH in HepG2 cells transfected with si-uc.333 or NC for 48 h, as analyzed using Western blot. Data are mean ± SEM; *P < 0.05; **P < 0.01; ***P < 0.001 vs. control group (Student’s t test).

To further investigate the role of uc.333 in IR, we transfected siRNA targeting uc.333 in HepG2 cells. RT-PCR showed that uc.333 was significantly inhibited in HepG2 cells transfected with si-uc.333 compared with HepG2 cells transfected with negative control (NC) (Figure 4A). We further examined the glycogen and glucose content in the HepG2 cells transfected with si-uc.333. Our data showed that the glucose level was increased after silencing uc.333, whereas glycogen contents were decreased after transfection of si-uc.333 in HepG2 cells (Figures 4B and 4C). We also determined the levels of AKT and GSK phosphorylation in the HepG2 cells transfected with si-uc.333 or NC. As shown in Figure 4D, inhibition of uc.333 reduced the levels of P-AKT and P-GSK.

### Interaction between uc.333 and miR-223

**Figure 5:**
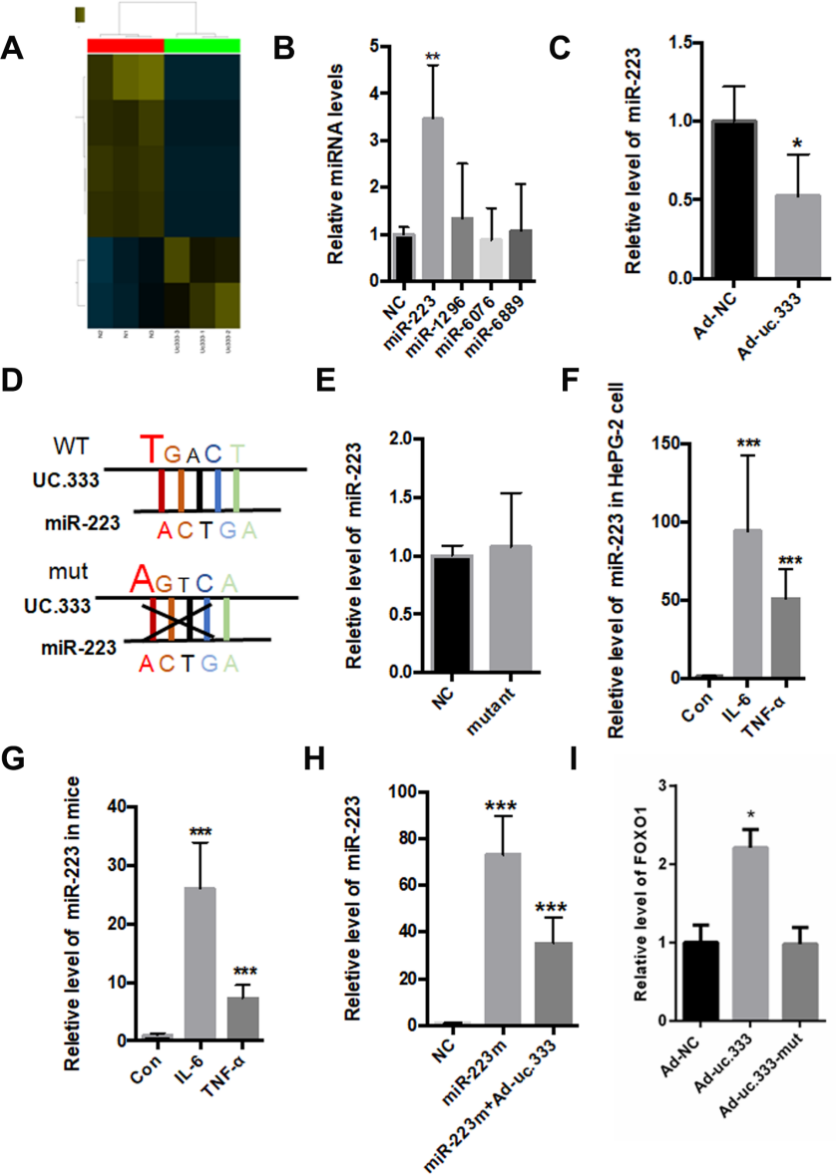
**Interaction between Uc.333 and miR-223.** (**A**) MiRNA-wide expression profiling in the HepG2 cells transfected with si-uc.333 (**B**) Expression of miR-223, miR-1296, miR-6067, and miR-6889 in the HepG2 cells transfected with si-uc.333 for 24 h. (**C**) Relative level of miR-223 in si-uc.333-infected HepG2 cells. (**D**) The sequence of miR-223 and its partial complementarity with uc.333. (**E**) Levels of miR-223 in the HepG2 cells transfected with Ad-uc.333 WT or Ad-NC mutant for 48 h. (**F**) Real-time PCR showed that miR-223 was decreased in HepG2 cells induced with IL-6 or TNF-α for 48 h. (**G**) Level of miR-223 in the livers of mice injected with IL-6 or TNF-α for 7 days. (**H**) Level of miR-223 in HepG2 cells transfected with miR-223 mimic with or without Ad-uc.333. (**I**) Tranfection with Ad-uc.333 significantly decreased the mRNA level of FOXO1, but Ad-uc.333-mut did not change the mRNA level of FOXO1 (Figure 5I). Data are mean ± SEM; *P < 0.05; **P < 0.01 vs. control group. (**C**, **E**: Student’s t test; **B**, **F**, **G**, **H**, **I**: analysis of variance [ANOVA]).

As an lncRNA, uc.333 is located in the cytoplasm, suggesting that uc.333 might exert its biological function in the cytoplasm. The correlation between miRNAs and UCRs has been widely reported [14]. To identify putative miRNA targets for uc.333, we overexpressed uc.333 in HepG2 hepatocytes and carried out a microarray analysis. A total of 5 miRNAs presented in the array were expressed at levels 1.5-fold or greater than the other miRNAs (Figure 5A). Next, we tested the expression of these miRNAs using quantitative RT-PCR and found that silence of uc.333 significantly increased the level of miR-223 (Figure 5B). As shown in Figure 5C, the miR-223 level was decreased when uc.333 was overexpressed in the HepG2 cells. Of note, miR-223 displayed noncomplementarity with the ultraconserved region of uc.333. To disrupt the interaction between uc.333 and miR-223, we constructed mutants in which the complementary nucleotides were deleted from uc.333 (Figure 5D). As expected, when the mutant form of uc.333 was overexpressed, no changes in miR-223 level were found (Figure 5E). Therefore, we analyzed the expression of miR-223 in the livers of mice injected with IL-6 and TNF-α. The expression of miR-223 was remarkably upregulated in the IL-6-treated and TNF-α-treated liver tissues compared with the corresponding normal liver tissues (Figure 5F). Additionally, we assessed the expression of miR-223 in human HepG2 hepatocytes stimulated with 10 nmol/L IL-6 for 24 h and 10 nmol/L TNF-α for 24 h. Interestingly, our data showed that the level of miR-223 was significantly increased in HepG2 cells stimulated by IL-6 and TNF-α (Figure 5G). To further evaluate whether uc.333 interacted with miR-223, we transfected a miR-223 mimic into HepG2 cells with or without Ad-uc.333. Our data showed that miR-223 was significantly decreased in the HepG2 cells transfected with Ad-uc.333, indicating that there is a relationship between miR-223 and uc.333 (Figure 5H). Furthermore, we tested the effect of UC.333 and its variant on the expression of FOXO1 (Figure 5I). Our data showed that transfection with Ad-uc.333 significantly decreased the mRNA level of FOXO1, but Ad-uc.333-mut did not change the mRNA level of FOXO1 (Figure 5I).

### miR-223 regulated IR through direct targeting of FOXO1

**Figure 6:**
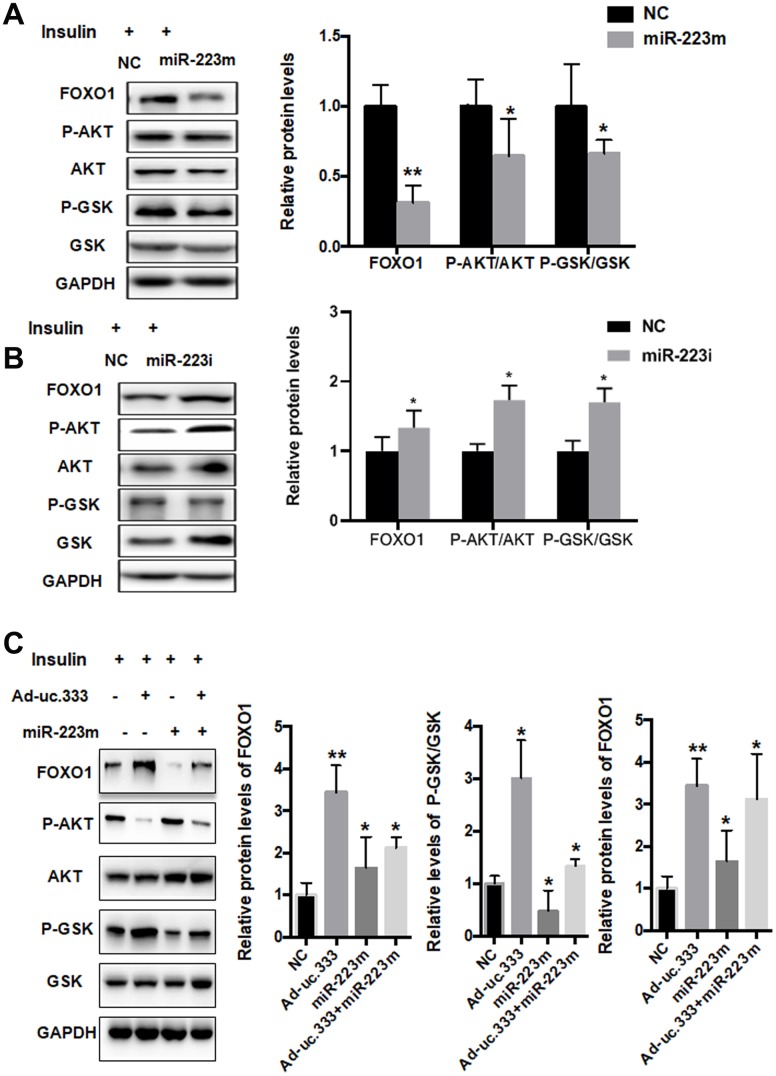
**miR-223 regulated IR through direct targeting of FOXO1.** (**A**) Protein levels of P-AKT, AKT, P-GSK, GSK, and GAPDH in HepG2 cells transfected with mimic miR-223 or NC for 48 h, as analyzed using Western blot. (**B**) Protein levels of P-AKT, AKT, P-GSK, GSK, and GAPDH in HepG2 cells transfected with miR-223 inhibitor or NC for 48 h, as analyzed using Western blot. (**C**) Protein levels of P-AKT, AKT, P-GSK, GSK, and GAPDH in HepG2 cells transfected with mimic miR-223 or NC for 48 h, as analyzed using Western blot. Data are mean ± SEM; *P < 0.05; **P < 0.01 vs. control group (Student’s t test).

Previous studies have shown that FOXO1 is a target gene of miR-223 [15, 16]. We overexpressed miR-223 in HepG2 cells using a specific miR-223 mimic, and analysis showed that the miR-223 mimic significantly suppressed the protein level of FOXO1 (Figure 6A). The phosphorylation levels of AKT/GSK were also decreased. To further investigate the role of miR-223 in IR, we silenced miR-223 with a miR-223 inhibitor. The results indicated that the miR-223 inhibitor increased FOXO1 expression and AKT/GSK phosphorylation levels (Figure 6B).

To investigate the effect of uc.333 on FOXO1 expression, we determined the levels of FOXO1, P-AKT, and P-GSK in the HepG2 cells transfected with the miR-223 mimic in the presence or absence of Ad-uc.333. Interestingly, we found that overexpression of uc.333 abolished the miR-223 mimic–induced downregulation of FOXO1, P-AKT, and GSK (Figure 6C), indicating that miR-223 is a key mediator in uc.333-mediated insulin sensitivity by regulating AKT/GSK/FOXO1 signaling.

### uc.333 expression was decreased in NAFLD

**Figure 7:**
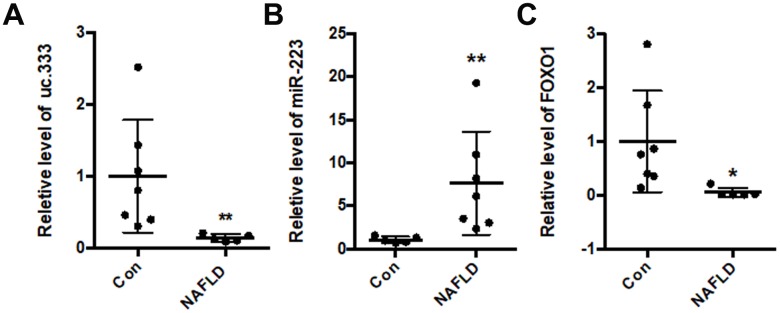
**uc.333 expression was decreased in NAFLD patients.** (**A**) Expression of uc.333 mRNA in the liver of NAFLD patients. (**B**) Identification of miR-223 in the liver of NAFLD patients. (**C**) Expression of FOXO1 mRNA in the liver of NAFLD patients. Data are mean ± SEM; *P < 0.05; **P < 0.01 vs. control group (Student’s t test).

Based on these findings, we determined the expression of uc.333 in the livers of NAFLD patients. Compared with that of the controls, the level of uc.333 was decreased in the livers of the NAFLD patients (Figure 7A). Moreover, the level of miR-223 was significantly increased in the livers of NAFLD patients (Figure 7B). The results from the RT-PCR assays showed decreased levels of FOXO1 mRNA in the livers of NAFLD patients (Figure 7C).

## DISCUSSION

ucRNAs are reported to be key regulators in both tumor oncogenesis and human cancer suppression [8]. Recently, we show that uc.372 is upregulated in the livers of NAFLD patients and is involved in lipid metabolism by affecting the maturation of miR-195 and miR-4668 [13]. However, the role of ucRNAs in IR remains unclear. In this study, we identified the downregulation of uc.333 in the livers of db/db mice, HFD-fed mice, IL-6-treated mice, TNF-α-treated mice, and NAFLD patients, indicating that uc.333 may play a key role in IR that is induced by inflammation factors. In vitro studies also showed that IL-6 and TNF-α significantly decreased the level of uc.333 in HepG2 cells. Moreover, overexpression of uc.333 significantly activated AKT/GSK signaling, whereas suppression of uc.333 decreased the level of P-AKT and P-GSK. It thus appears that uc.333 improves glucose metabolism via regulation of PI3K/AKT/GSK signaling.

Intronic ultraconserved regions are often related to the regulation of transcription and DNA binding, implying their potential role as regulators of gene expression [8, 11]. For example, one study indicated that uc.134 represses hepatocellular carcinoma development by inhibiting CUL4A-mediated ubiquitination of LATS1 and that uc.416 inhibits miR-153 and promotes epithelial-to-mesenchymal transition in renal cell carcinoma [10, 12]. Besides, uc.283+ is shown to inhibit pri-miR-195 recognition and cropping [21]. And uc.416 is shown to suppress miR-153 expression and induces epithelial-to-mesenchymal transition in renal cell carcinoma [12]. As an intronic exon-containing lncRNA that is enriched in the cytoplasm, whether uc.333 could bind to miRNAs with the structural similarity of lncRNAs and miRNAs remain unknown. In the present study, miRNA microarray analysis showed that inhibition of uc.333 significantly increased miR-223 level. Previous studies have shown that miR-223 could be involved in a variety of biological activities such as tumor suppression or metabolic disease [22–27]. Furthermore, it is reported that miR-223 knockout mice exhibit impaired glucose tolerance and IR [20]. Here, in vitro studies validated that uc.333 can suppress the level of miR-223 in HepG2 cells. These observations indicate that uc.333 may function as a sponge for miR-223. Bioinformatic analysis has shown that the structure of miR-223 has an imperfect pair region between miR-223 and uc.333. Moreover, the expression of miR-223 was dramatically increased in the livers of HFD-fed mice, IL-6-injected mice, TNF-α mice, and NAFLD patients. Therefore, we propose that uc.333 is involved in the progression of IR via binding miR-223.

A recent study reports that miR-223 blunts the compensatory response of β-cells to HFD-induced IR by directly targeting FOXO1 [15]. FOXO1 is a member of the FOX transcription factors O subfamily, which is characterized by a totally conserved forkhead domain [17, 18]. Evidence suggests that FOXO1 is essential during the development of IR because of its inhibition faction in glucose uptake, and that miR-223 may suppress its expression [17, 19]. In line with previous studies, we found that miR-223 markedly suppressed the mRNA level of FOXO1, and more importantly, overexpression of uc.333 reversed miR-223-mediated IR in HepG2 cells, indicating that uc.333 improves IR via miR-223-induced suppression of FOXO1/AKT/GSK signaling.

However, there are limitations in the present study. First, it is interesting to explore the effect of UC.333 and its mutant in the activity of reporters bearing the FOXO1 3'UTR, which directly elucidate the interaction between uc.333 and FOXO1. Secondly, we have shown that the expression of uc.333 was also dramatically decreased in the fat and pancreas organs, which are also closely related to IR progression. But the specific underlying mechanism has not been elucidated, whether they share the same molecular mechanism or not in these organs deserves further study. In the future, we will conduct a detailed study to elucidate the role of uc.333 in the fat and pancreas tissues. In summary, for the first time, we showed novel data that uc.333 improves IR by binding to miR-223; thus, uc.333 may be a useful target for the treatment and prevention of IR.

## MATERIALS AND METHODS

### Human liver specimens

Liver biopsies were performed on 8 NAFLD patients and 8 control subjects. The application for patient-derived materials was approved by the Research Ethics Committee of Beijing You-An Hospital (BJYSE2015-35), and written consent was obtained from all patients.

### Experimental animals

The experimental animals included 8-week-old male db/db mice and 8-week-old C57BL/6 mice purchased from Peking University Health Science Center. C57BL/6J mice were subcutaneously injected with 16 μg/mL^–1^ IL-6 and 16 μg/mL^–1^ TNF-α using Akzet osmotic pumps (Durect, Cupertino, CA, USA) for 7 days according to previous description, which demonstrated the successful construction of these animal models for IR [28]. and another group of mice were fed a chow diet or high-fat diet (HFD, containing 21% fat, 19.5% casein, and 1.25% cholesterol) to construct an NAFLD model, as described previously [13]. All animal experiments conformed to the protocols approved by the Animal Use and Care Committee of Beijing Hospital and the Guide for Care and Use of Laboratory Animals (NIH Publication #85-23, revised 1996).

### Cell culture

HepG2 human hepatic carcinoma cell line was obtained from the American Type Culture Collection (ATCC), and the cells were cultured in Eagle’s minimum essential medium (MEM; Invitrogen, Carlsbad, CA, USA) with 10% fetal bovine serum (FBS; HyClone, Logan, UT, USA), 100 U/mL penicillin (Invitrogen), 0.1 mg/mL streptomycin (HyClone), and 1 mm sodium pyruvate at 37°C with humidified air and 5% CO_2_.

### Construction of adenoviral vectors

Adenoviral vectors overexpressing uc.333 mimic (Ad-uc.333) and control vector (Ad-NC) were constructed by Genepharma (Shanghai, China). The adenovirus vectors were transfected into HepG2 cells at the density of 100 multiple of infection (MOI). Transfection efficiency was detected under a Zeiss Axio Observer Z1 Apotome fluorescence microscope.

### Transient transfection

Si-uc.333 and nonspecific siRNA (NC) were constructed by Genepharma. Transfection of siRNAs was performed with HiPerFect transfection reagent (Qiagen, Duesseldorf, Germany) according to the instructions. Briefly, 6 × 10^5^ cells were seeded in 6-well plates with 2 mL MEM culture medium containing 10% FBS and antibiotics. At the same time, siRNAs or NC was mixed with HiPerFect transfection reagent and incubated at room temperature for 10 min. Then, the complexes were transfected into HepG2 cells for 48 h.

### miRNA array analysis

A total of 20 μg of RNA was isolated from HepG2 cells transfected with Si-uc.333 and NC. Total RNA was reverse transcribed with random oligonucleotide primers. The transcriptome for the mouse lncRNA was analyzed using an Agilent mouse lncRNA + mRNA Array V1.0 (CapitalBio, Beijing, China), including sense and antisense probes for all 481 human ultraconserved sequences, each of which was spotted in duplicate. For RNA isolated from HepG2 cells, the transcriptome for human miRNA was analyzed using Agilent human miRNA Microarray V21.0 (CapitalBio).

### Western blot analysis

The liver tissues and cells were extracted with RIPA buffer (CST). Immunoblotting for AKT, GSK, FOXO1, and GAPDH was performed using established procedures. [14] Bands were visualized using a chemiluminescence detection system (Vilber Lourmat, Collégien, France) according to previous description [13].

### RNA isolation and quantitative PCR validation

Total RNA from HepG2 cells and liver samples from db/db mice, IL-6-treated mice, TNF-α-treated mice, and NAFLD patients were isolated with TRIzol reagent (Invitrogen, Carlsbad, CA, USA) and then reverse transcribed using a reverse transcription system kit (NEB, M-MLV kit). The relative mRNA levels were quantified using SYBR Green PCR Master Mix and analyzed with a Bio-Rad real-time PCR detection system. GAPDH was used as internal control. The relative expression levels were calculated with the 2-ΔΔCt method, and the experiments were repeated in triplicate. The primers used in the current study are listed in Supplementary Table 1.

### RNA fluorescence in situ hybridization

The probes to uc.333 RNA and U6 were designed following the Stellaris RNA fluorescence in situ hybridization probe designer (Biosearch Technologies). Cell fixation, permeabilization, and hybridization to probes were performed following protocols for adherent cells. Images were acquired with a Zeiss Axio Observer Z1 Apotome fluorescence microscope.

### Glycogen content measurement

After the cells were treated for 24 h, the culture solution was carefully discarded. Then the cells were washed once with PBS, and the cells were lysed with RIPA lysate. After that, the intracellular glycogen content was measured with a glycogen kit (Biovision, Mountain View, CA, USA) according to the manufacturer’s instructions.

### Glucose content test

After the cells were treated for 24 h, 50 μL of the supernatant of the cells to be tested was taken, and the glucose content was determined using a glucose kit (Bioassay Systems DIGL-100). The results were expressed as a percentage of the glucose content of the model group.

### Cell treatment

To evaluate the effect of uc.333 and miR-223, HepG2 cells were cultured with or without TNF-α (15 nmol/L), IL-6 (10 nmol/L) for 24 h, and 33.3 mmol/L glucose for 48 h. HepG2 cells were also treated with Oleic acid/palmitic acid (O/P) as previously described [13]. Then they were tested or treated with insulin (100 nM) for 15 min for the next assays.

### Statistical analysis

The data are presented as the mean ± standard error of the mean. Comparisons between groups were made using a 2-tailed Student’s t test. For comparisons of multiple groups, 1-way analysis of variance was used. *P* values less than 0.05 were considered significant.

## Supplementary Material

Supplementary Table 1
