# Supplementary material for: Ultraconserved element uc.333 increases insulin sensitivity by binding to miR-223
Source: Aging (Albany NY). 2020 Apr 17;12(8):6667–79. doi: 10.18632/aging.103020 (PMC7202487; doi:10.18632/aging.103020)
Supplement: Supplementary Table 1 [file aging-12-103020-s001..pdf]

SUPPLEMENTARY TABLE

Supplementary Table 1. A list of real-time PCR primers used.

Gene Name	Forward Primer	Reverse Primer
uc.333	ACTGCTCTTTCTTTTCCCAA	TTTGTGTCTTAGACCCTTCG
histone	GAAGTCCACTGAACTGCTTA	CCTCAAATAGCCCTACCAAG
GAPDH	GTCGTGTGAACGGATTTG	AAGATGGTGATGGGCTTCC
U6	CTCGCTTCGGCAGCACA	CGGCTTCGGCTCTTAGCAAA
FOXO1	TCGTCATAATCTGTCCCTACACA	CATTTAAGACACTTACATCGCTA
miR-223	GCGCTGTCAGTTTGTCAAATACCCCA	GTGCAGGGTCCGAGGT
miR-1296	GCGCGCTTAGGGCCCTGGCTC	GTGCAGGGTCCGAGGT
miR-6076	GCGCAGCATGACAGAGGAGAGGTGG	GTGCAGGGTCCGAGGT
miR-6789	GCGCTCTGTGCCCCTACTTCCCAG	GTGCAGGGTCCGAGGT
uc.46	AATCAACCCACAAAGCTTCT	AATCAACCCACAAAGCTTCT
uc.443	GGAAACGTTATTGGTTCAGC	GGAAACGTTATTGGTTCAGC
uc.184	GGAAACGTTATTGGTTCAGC	GGAAACGTTATTGGTTCAGC
uc.193	GGAAACGTTATTGGTTCAGC	GGAAACGTTATTGGTTCAGC
uc.366	GGAAACGTTATTGGTTCAGC	GGAAACGTTATTGGTTCAGC
uc.419	GACAATAGCACTTGGAGTCA	GACAATAGCACTTGGAGTCA
uc.418	GACAATAGCACTTGGAGTCA	GACAATAGCACTTGGAGTCA
uc.36	GTGAGTGCAAGCAGTTTTAC	GTGAGTGCAAGCAGTTTTAC
uc.336	TGGAGGCAGACACAAAATTT	TTTCGCTCCCAATGAACATA
uc.185	CCGGCACATGACTCAGAAGG	CCGGCACATGACTCAGAAGG
uc.336	TGGAGGCAGACACAAAATTT	TGGAGGCAGACACAAAATTT
uc.353	CTTTCATTGTGCAGAGTGTG	CTTTCATTGTGCAGAGTGTG
uc.471	AAGGAAAGAATAGGGCACAG	AATGTACAAGGGCCTAAACC
uc.186	AGTGACATATCCAACCAACC	AAGGAAAGAATAGGGCACAG
uc.420	AGCTACTCTTTGCAATATGAAA	AGCAGTTCTTCCAATTCGAT
